# Mesenchylmal Stem Cell Culture on Poly(*N*-isopropylacrylamide) Hydrogel with Repeated Thermo-Stimulation

**DOI:** 10.3390/ijms19041253

**Published:** 2018-04-21

**Authors:** Aya Mizutani Akimoto, Erika Hasuike Niitsu, Kenichi Nagase, Teruo Okano, Hideko Kanazawa, Ryo Yoshida

**Affiliations:** 1 Department of Materials Engineering, School of Engineering, The University of Tokyo, 7-3-1 Hongo, Bunkyo, Tokyo 113-8656, Japan; 2 Faculty of Pharmacy, Keio University, 1-5-30 Shibakoen, Minato, Tokyo 105-8512, Japan; hasu.eri523@gmail.com (E.H.N.); kanazawa-hd@pha.keio.ac.jp (H.K.); 3 Institute of Advanced Biomedical Engineering and Science, Tokyo Women’s Medical University, 8-1 Kawada-cho, Shinjuku, Tokyo 162-8666, Japan; nagase-kn@pha.keio.ac.jp (K.N.); tokano@twmu.ac.jp (T.O.)

**Keywords:** poly(*N*-isopropylacrylamide), hydrogel, mesenchymal stem cell, cell culture, mechanobiology

## Abstract

We prepared thermoresponsive hydrogels by mixing poly(*N*-isopropylacrylamide) (PNIPAAm) derivatives as the main chain components, octa-arm polyethylene glycol (PEG) as a crosslinker, and the Arg-Gly-Asp-Ser (RGDS) peptides as cell adhesion units. Human bone marrow-derived mesenchymal stem cells (hbmMSCs) were cultured on the hydrogels. The PNIPAAm gel prepared by the post-crosslinking gelation method was revealed to be cytocompatible and showed temperature-dependent changes in mechanical properties. Repeated changes in the swelling ratio of the PNIPAAm gel affected the shape of the hbmMSCs. With respect to both cytocompatibility and reversibility of changes in mechanical properties, the PNIPAAm gel system could be potentially useful for the analysis of cell mechanobiology.

## 1. Introduction

In an organism, the function and fate of cells are controlled by signals from soluble factors such as cytokines and growth factors, contiguous cells, and extracellular matrices [1]. To mimic these signals *in vitro* for the development of artificial microenvironments for cell fate manipulation, a beneficial strategy is to design artificial extracellular matrices that can provide the appropriate physical and biochemical cues. Because extracellular matrices are mainly composed of proteins, sugars, and a large amount of water, their function can be partly substituted by synthetic soft materials such as polymer-coated surfaces, hydrogels, and fibers [2,3,4]. Thus, novel designs of soft materials have greatly influenced the progress of *in vitro* cell fate manipulation technologies. The pioneering study on soft materials technology was published by Ingber et al., in 1997 [5]. In this study, the adhesion areas of capillary endothelial cells were successfully controlled using glass surfaces on which fibronectin was adsorbed in a micro-pattern, revealing that cell survival and death by apoptosis are determined by the size of the adhesion areas. Through this study, materials engineering was revealed to have a great impact on the cell fate control research.

After roughly ten years, Engler et al., reported that nerve mesenchymal stem cells (MSCs) could be cultured on several types of polyacrylamide gels that have different elasticities [6]. This study made a large impact on research in materials engineering and biology because it demonstrated that the elasticity of cell culture substrates directs the differentiation lineage of MSCs. As a result of this finding, the use of synthetic soft materials to control cell fate has become more widespread, and it has led to numerous related studies [7,8,9]. However, these studies lacked information about dynamic cell behavior; the cell culture experiments in these studies had been conducted under static conditions, i.e., cells were separately cultured on several types of materials that have different physicochemical properties.

The study of cellular response under dynamic conditions is important because the microenvironment around cells inside the body is constantly changing. This environment is completely different from that of static cell cultures. Therefore, the development of functional soft materials that mimic dynamic microenvironmental changes is recognized as an urgent task. For this purpose, stimuli-responsive hydrogels are one of the most promising materials because their physical properties can be changed in situ, similar to living tissues. Recently, a class of stimuli-responsive hydrogels for cell culture, whose mechanical properties can be controlled in situ by pH and light have been reported [10,11,12]. The study conducted by Yang et al., using photo-degradable polyethylene glycol (PEG) gels is worthy of special mention because it demonstrated that human bone marrow-derived MSCs (hbmMSCs) can memorize the mechanical stimulus received from the cell culture material in the past and alter their fate according to the memory [12]. However, an optimal material that minimizes cytotoxicity and retains reversibility in changes in properties is not yet available.

To develop such a system, we cultured hbmMSCs using thermoresponsive poly(*N*-isopropylacrylamide) (PNIPAAm) hydrogels to repeatedly control the mechanical properties triggered by moderate, biocompatible temperature changes. PNIPAAm exhibits thermoresponsive coil-to-globule transition in aqueous solutions at approximately 32 °C, which is its lower critical solution temperature (LCST) [13]. This structural change is brought about by the reversible hydration/dehydration properties of its isopropyl side chains. Below the LCST, hydration allows the polymer to expand its chains in water, while above the LCST, dehydration results in an aggregated structure. Thus, when PNIPAAm is crosslinked to form a hydrogel, it reversibly swells and shrinks across a temperature close to the LCST, called the volume phase transition temperature. In this study, the thermoresponsive hydrogel was prepared by mixing PNIPAAm derivatives as the main chain components, octa-arm polyethylene glycol (PEG) as a crosslinker, and the RGDS peptide as a cell adhesion unit. hbmMSCs were cultured on the hydrogel. The mechanical properties of the hydrogel were repeatedly changed during hbmMSC culture, and cytotoxicity and cellular response to changes in the dynamic properties of the hydrogel were analyzed.

## 2. Results

### 2.1. Characterization of Hydrogels

The colorless and transparent PNIPAAm gel was obtained by mixing PNIPAAm derivatives (main chain polymer, providing amino moieties), *N*-hydroxysuccinimide-terminated octa-branched polyethylene glycol (NHS-octa-PEG) (crosslinker polymer), and RGDS peptides, in the so-called post-crosslinking method [14]. This method is advantageous for cell culture applications because the gelation process does not contain harmful compounds such as monomers, and the procedure makes it easy to prepare gels inside cell culture dishes or wells of micro-plates. Poly(*N*,*N*-dimethylacrylamide) (PDMAAm) gels, which do not exhibit thermoresponsiveness, were prepared by the same method as controls.

**Figure 1:**
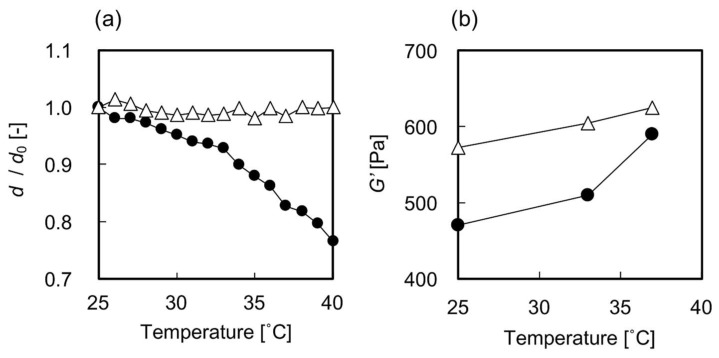
(**a**) The swelling ratio and (**b**) *G’* of gels relative to temperature change. Symbols: closed circles, PNIPAAm gel; open triangles, PDMAAm gel.

First, the temperature-dependent swelling ratios of the gels were measured by optical microscopic observation. As shown in Figure 1a, the swelling ratios of the PNIPAAm gel decreased as the temperature increased because of the thermoresponsive conformational change in PNIPAAm, while the swelling ratios of the PDMAAm gel were constant throughout the measurement. Volume changes of roughly 10% were observed in the PNIPAAm gel in the temperature range of 33–37 °C. This means that the swelling ratios of the PNIPAAm gel could be easily controlled by moderate temperature changes in the range of physiological temperatures. The storage elastic modulus (*G’*) of the gels was measured using a rheometer. The *G’* of PNIPAAm gels was shown to be temperature-dependent, which seemed to be attributed to the thermoresponsive conformational change in PNIPAAm (Figure 1b). However, the *G’* of the PDMAAm gels also showed a slight temperature dependency. This may be caused by the temperature-dependent shrinkage of the network structure caused by the rubber elasticity of the hydrogel.

**Figure 2:**
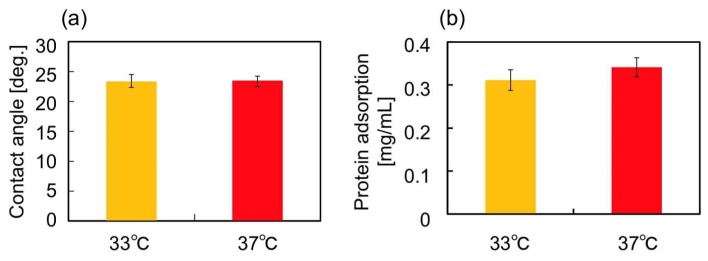
(**a**) Contact angles and (**b**) amount of serum protein adsorption on the PNIPAAm gel at 33 and 37 °C. Data are expressed as the mean of three measurements (± standard deviation).

Next, surface wettability and the amount of deposited proteins on the gel surfaces were analyzed by the measurement of contact angles and the micro BCA^TM^ protein assay, respectively at 33 and 37 °C. Such surface characteristics strongly affect on cell adhesion [2]. As shown in Figure 2, the changes in these surface characteristics were revealed to be negligible. Thus, surface characteristics of these two gels can be recognized as equivalent.

### 2.2. Cytotoxicity Assessment

**Figure 3:**
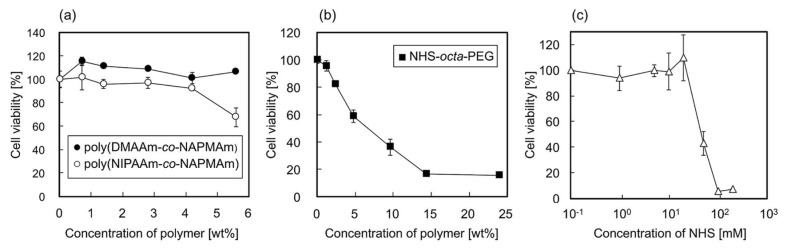
Relative viability of hbmMSCs exposed to (**a**) poly(DMAAm-*co*-NAPMAm) and poly(NIPAAm-*co*-NAPMAm), (**b**) NHS-octa-PEG, and (**c**) NHS for 18 h at 37 °C, compared to that of cells without exposure. Data are expressed as the means of triplicate measurements (± standard deviation).

The cytotoxicity of the hydrogel components and the side products of the gelation reaction were evaluated using hbmMSCs. Analysis of concentration-dependent cytotoxicity of the main chains of the hydrogels, copolymers of NIPAAm and *N*-3-(aminopropyl) methacrylamide (NAPMAm) (poly(NIPAAm-*co*-NAPMAm)), and poly(DMAAm-*co*-NAPMAm), shown in Figure 3a, revealed that at 2.8 wt %, which is the concentration used in the gelation reaction, the polymers did not show any apparent cytotoxicity after hbmMSCs were exposed to the polymers for 18 h. In contrast, exposure of hbmMSCs to 3.0–4.8 wt % NHS-octa-PEG decreased the viability by 20–40% (Figure 3b). This could be explained by the reaction between the activated ester moieties of NHS-octa-PEG and the amino moieties on the surface of the hbmMSCs. However, in the practical experiments, when the gels are used for cell culture, many of the activated ester moieties originating from NHS-octa-PEG would have reacted with the main chains and RGDS peptides. Therefore, the cytotoxicity of NHS-octa-PEG should be greatly decreased when the gels are used for cell culture experiments. This is also supported by the LIVE/DEAD staining of hbmMSCs cultured on the PNIPAAm gel (Figure S1). As shown in Figure S1, almost all hbmMSCs were alive even after they had been cultured inside the PNIPAAm gel for seven days. Furthermore, NHS, as the side product of the reaction between activated ester moieties and amino moieties, was suggested to have no effect on cytotoxicity under conditions used in the gelation reaction (Figure 3c). From these results, the gelation reaction can be considered to be cytocompatible, and the PNIPAAm and PDMAAm gels suitable cell culture substrates. In summary, the in situ gelation by the post-crosslinking reaction enables the easy preparation of cytocompatible gels inside cell culture dishes or plates. The preparation does not require purification processes and only requires medium changes.

### 2.3. Cellular Response to Dynamic Mechanical Stimuli

**Figure 4:**
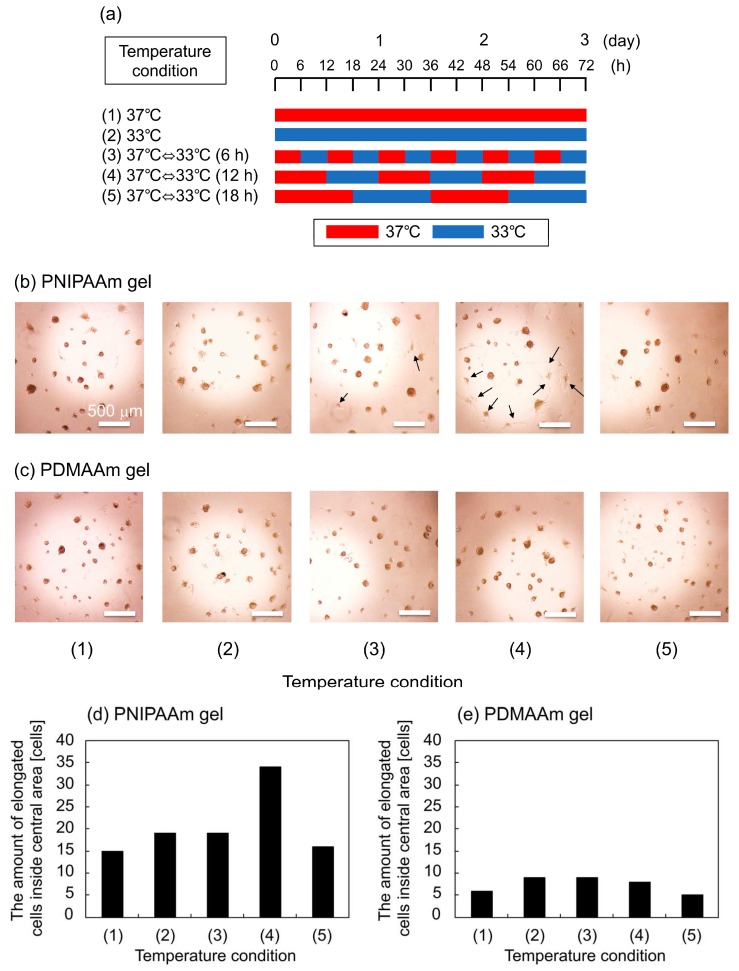
(**a**) Schematic explanation of temperature conditions for the cell culture experiments. Phase-contrast images of hbmMSCs on (**b**) PNIPAAm gel and (**c**) PDMAAm gel after three days in culture using AIM as cell culture media. The amount of elongated cells inside central area of (**d**) PNIPAAm gel and (**e**) PDMAAm gel after three days in culture using AIM as cell culture media. The arrows in the panel (**b**) shows elongated cells.

The effect of thermoresponsive changes in the mechanical properties of the PNIPAAm gel as a cell culture substrate on the adipogenic induction of hbmMSCs was evaluated. When hydrogels are used as cell culture substrates, a storage elastic modulus of several hundred pascals (Pa) is known to be a good condition for adipogenic induction of hMSCs [15]. Since the elastic moduli of our PNIPAAm gels were in this range, we chose hbmMSCs as the target cell type. We applied two static temperature conditions, denoted as (1) and (2), and three types of rhythmical temperature changes between 33 and 37 °C, denoted as (3)–(5), to gels with hbmMSCs to evaluate the effect of the periodicity of mechanical stimulus (Figure 4a). Considering that the metabolic activity of a cell depends on temperature, we set the physiological temperature, 37 °C, as the cell culture temperature, and used another temperature, 33 °C, which is close to the physiological temperature. Figure 4b–e shows the phase-contrast microscopic images of hbmMSCs and the amount of elongated hbmMSCs, respectively on the gels after three days of incubation in adipogenic induction medium (AIM) with the five temperature conditions. The results showed that larger numbers of hbmMSCs were deformed on the PNIPAAm gel compared with those on the PDMAAm gel. In particular, in condition (4), the number of elongated hbmMSCs on the PNIPAAm gel was high. Pseudopodia were observed on hbmMSCs adhering on the RGDS sites of the PNIPAAm gel in conditions (3) and (4). This indicates that repeated property changes of the PNIPAAm gel in 6- and 12-h cycles altered the shape of hbmMSCs, compared with property changes in other conditions.

### 2.4. Evaluation of Adipocyte Differentiation

We evaluated the amount of accumulated lipid droplets inside hbmMSCs on the gels using the AdipoRed^TM^ assay after incubation with five temperature conditions in AIM for three days and subsequent incubation in maintenance media for four days, i.e., a total of seven days of incubation. As a result, there was no significance found in the statistical analysis between the five conditions.

## 3. Discussion

The most plausible factor that induced the elongation of hbmMSCs grown in the PNIPAAm gel could be the repeated in situ changes in the swelling ratios. We speculate that this is the case because the swelling ratio showed the largest difference between the PNIPAAm and the PDMAAm gels. Although the *G’* of the PNIPAAm gel also showed temperature-dependent changes, it was quite difficult to differentiate the effect of thermoresponsive conformational changes of PNIPAAm gel compared with the that of the PDMAAm gel. Moreover, the difference between the *G’* values measured at 33 and 37 °C, which was around 100 Pa, was significantly smaller than that reported in previous studies which described the effects of *G’* in cell culture substrates on the shapes of adhered cells [16] and the adipogenic differentiation of hMSCs [15,17]. Additionally, we confirmed that there were no significant differences between the surface contact angle as well as the amount of adsorbed serum proteins on the PNIPAAm gel at 33 and 37 °C (Figure 2a,b). The effect of the temperature change itself can also be considered negligible based on the lack of difference in cell shape observed on the PDMAAm gel (Figure 4c). Thus, it can be concluded that the repeated changes in the swelling ratio, i.e., the pushing and pulling force on hbmMSCs in the PNIPAAm gel enabled cell stretching and altered the shape of the hbmMSCs. It is still unclear why the 12-h cycle was especially effective among the conditions (3)–(5). Considering the difference among them, frequency of temperature change and time of cell culture at each temperature could have potentially affected cell behavior, cell cycle, and mechanical memory of hbmMSCs [12].

**Figure 5:**
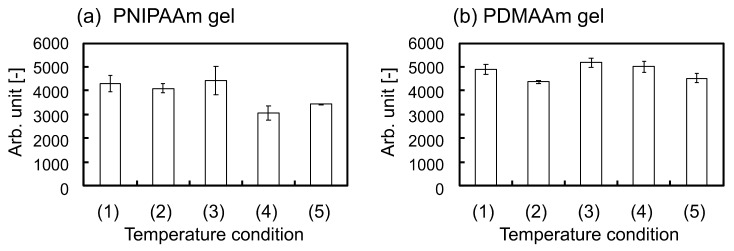
Fluorescence intensity showing the amount of accumulated lipid droplets inside cells obtained from the AdipoRed^TM^ assay on (**a**) PNIPAAm gel and (**b**) PDMAAm gel. (1)–(5) show the temperature conditions for cell culture as explained in Figure 4. Data are expressed as the mean of three measurements (± standard deviation).

On the other hand, when hbmMSCs differentiate into adipocytes, shapes of cells are changed from elongated to round; elongation implies undifferentiated state [15,17]. It is also clarified by Figure S2 that many hbmMSCs cultured on the PNIPAAm gel by using MSCBM, normal cell culture medium, showed elongated shapes. Thus, our results suggest that adipogenic induction of hbmMSCs cultured in AIM is suppressed by specific changes in the swelling ratio of the PNIPAAm gel. This hypothesis is also supported by a previous study by Tanabe et al. [18]. Adipoprogenitor cells were cultured on a silicone-stretching chamber and it was revealed that repeated stretching forces suppressed adipogenic induction. If the adipogenic induction of hbmMSCs on the PNIPAAm gel was suppressed by repeated thermo-stimulation, the phenomenon is potentially governed by the similar signal transduction pathway identified previously [18]. To elucidate the mechanism, we evaluated the amount of accumulated lipid droplets inside hbmMSCs on the gels using the AdipoRed^TM^ assay after incubation with five temperature conditions. The results showed that adipogenic induction seemed to be slightly restricted by repeated thermo-stimulation of the PNIPAAm gels (Figure 5). However, the differences in the amounts of deposited lipid droplets inside the cells between the five conditions were quite small (no significance was found in the statistical analysis). To enable more effective cell fate manipulation, it is necessary to improve the design of the gel. Modified designs of PNIPAAm gels, such as those with porous structures and aggregated micro-gel structures which would enhance thermoresponsive changes in the swelling ratio, are currently under study.

## 4. Materials and Methods

### 4.1. Materials

*N*-isopropylacrylamide (NIPAAm) was kindly provided by KJ Chemicals (Tokyo, Japan) and purified by recrystallization in a mixture of hexane: toluene 90:10 (*v*/*v*). *N*-3-(aminopropyl) methacrylamide (NAPMAm) was purchased from Polysciences (Washington, PA, USA). *N*-hydroxysuccinimide-terminated octa-branched polyethylene glycol (NHS-octa-PEG, 20 kDa, SUNBRIGHT HGEO-200SH) was purchased from NOF Co., Ltd. (Tokyo, Japan). RGDS peptides were purchased from Bachem AG (Budendorf, Switzerland). Mesenchymal Stem Cell Basal Medium (MSCBM), Mesenchymal Stem Cell Growth Medium SingleQuots^TM^ Supplements and Growth Factors, AdipoRed™ Assay Kit, and human bone marrow-derived mesenchymal stem cells (hbmMSCs) were purchased from Lonza Japan (Tokyo, Japan). Mesenchymal Stem Cell Adipogenic Differentiation Medium 2 (adipogenic induction medium, AIM) was purchased from Takara Bio (Shiga, Japan). Micro BCA™ Protein Assay Kit was purchased from Thermo Fisher Scientific (Waltham, MA, USA). LIVE/DEAD Viability/Cytotoxicity Kit (L3224) was purchased from Life Technologies (Gaithersburg, MD, USA). All other reagents were purchased from Wako Pure Chemical Industries (Osaka, Japan) and used as received.

### 4.2. Synthesis of Hydrogels

**Figure 6:**
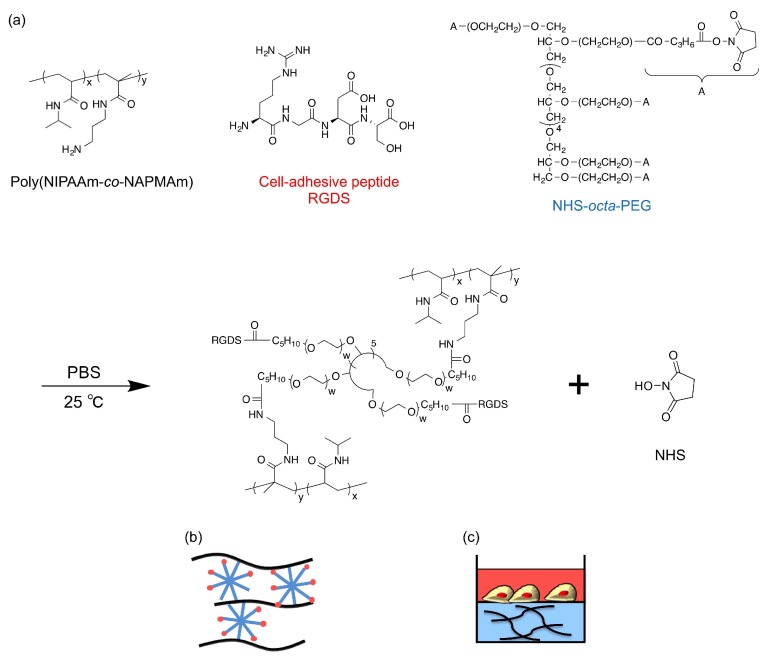
(**a**) Scheme for the gelation of the PNIPAAm gel. Schematic illustrations of (**b**) the molecular structure of the PNIPAAm gel and (**c**) the experimental set-up for cell culture on the gels.

PNIPAAm hydrogels and PDMAAm hydrogels as a control were synthesized by the post-crosslinking method slightly modified from our previous study [14]. First, NIPAAm (or DMAAm) and NAPMAm were copolymerized by free radical polymerization (molar ratio of NIPAAm or DMAAm:NAPMAm = 98:2). The prepared poly(NIPAAm-*co*-NAPMAm) (28 mg), NHS-octa-PEG (48 mg) as a crosslinker, and RGDS peptides (3.0 mg) as cell-adhesive molecules were simply mixed in 1 mL of PBS (pH 7.4) and incubated for 5 min at 25 °C to synthesize PEG-crosslinked PNIPAAm gels with RGDS pendants (PNIPAAm gel) (Figure 6). Poly(DMAAm-*co*-NAPMAm) (28 mg), NHS-octa-PEG (32 mg), and RGDS peptides (3.0 mg) were similarly mixed in 1 mL of PBS (pH 7.4) to obtain PEG-crosslinked PDMAAm gels with RGDS pendants (DMAAm gel) as a control.

### 4.3. Characterization of the Hydrogels

The temperature-dependent swelling ratios of the PNIPAAm gel and PDMAAm gel (0.63 mmØ cylindrical gel) whose solvent was MSCBM were measured by optical microscopic observation (MZ16, Leica, Mannheim, Germany). A cylindrical-shaped gel was equilibrated in MSCBM for 1 h at each temperature in the range of 25–40 °C before measurement. The swelling ratio was defined as the diameter of the gel normalized to the inner diameter of the glass capillary (d_0_ = 0.63 mm), which was used as a mold during the gelation reaction.

Measurements of the storage elastic modulus (*G*’) of the sheet-shaped PNIPAAm gel and PDMAAm gel whose solvent was MSCBM were performed on an Anton Paar Physica MCR 301 rheometer (Anton Paar, Graz, Austria). Parallel-plate geometry was employed using a 25-mm diameter plate with a constant deformation of 2% and a frequency of 1.0 Hz. The gel was kept at 33 °C or 37 °C for 20 min, and a normal force was kept at approximately 0.3 N for all measurements.

Thermoresponsive surface wettability of the sheet-shaped PNIPAAm gel was evaluated by static contact angle measurements (a drop-shape analyzer, DSA100, KRÜSS, Hamburg, Germany) using the captive bubble method and analyzed by the tangent-1 method. The PNIPAAm gel sheet was immersed in Milli-Q water in quartz cells and contact angle was measured after placing an air bubble onto the sample surface. The sample temperature was regulated at 33 or 37 °C. Data are expressed as the mean of three measurements with standard deviation.

Temperature-dependent protein adsorption on the PNIPAAm gel was evaluated using the Micro BCA™ method. The PNIPAAm gel (150 μL/well) was prepared inside the wells of a 24-well microplate and MSCBM (300 μL/well) was poured into the wells. After incubation for 60 min at 33 or 37 °C, the gel was washed five times with PBS (400 μL/well), and 5 wt % sodium dodecyl sulfate (600 μL/well) was added and incubated for 60 min at 25 °C to recover the adsorbed serum proteins from the surface of the gel. According to the protocol of the Micro BCA™ Assay Kit, the total amounts of adsorbed serum proteins on the gel at each temperature were quantified.

### 4.4. Cytotoxicity Assessment

The cytotoxic effects of adding polymer components of the gel and *N*-hydroxysuccinimide (NHS), as a side product of gelation, on cell culture medium were assessed by evaluating the viability of hbmMSCs, which were cultured on conventional tissue culture polystyrene (TCPS) dishes in MSCBM at 37 °C in a humidified atmosphere with 5.0% CO_2_. The hbmMSCs were recovered from the TCPS dishes via treatment with D-PBS containing trypsin and ethylenediaminetetraacetic acid. The cells recovered in MSCBM were seeded onto a white 96-well microplate at 5.0 × 10^4^ cells/cm^2^ and incubated at 37 °C for 3 h in a humidified atmosphere with 5.0% CO_2_. Then, PBS containing poly(NIPAAm-*co*-NAPMAm), poly(DMAAm-*co*-NAPMAm), NHS-octa-PEG, or NHS was poured onto the surfaces and incubated for 18 h at 37 °C. After the incubation, the cell viability was measured with the general procedure using the acetomethoxy derivative of calcein, which is hydrolyzed by intracellular esterases to produce a strong yellow green fluorescence (*λ*_ex_ = 485 nm, *λ*_em_ = 535 nm). The cell viability was analyzed by comparing compound-exposed cells to unexposed cells as a control.

### 4.5. Cell Culture

The effects of temperature-dependent changes in the mechanical property of the PNIPAAm gel on the shape of hbmMSCs were investigated during the adipogenic induction of hbmMSCs on the PNIPAAm gel. PNIPAAm gels or PDMAAm gels (50 μL/well) were prepared inside the wells of a 96-well microplate and the solvent was changed from PBS to AIM. hbmMSCs suspended in AIM (100 μL containing 1.0 × 10^4^ cells/well) were seeded on the gels and cultured for three days in a humidified atmosphere with 5.0% CO_2_. During cell culture in AIM, the temperature was repeatedly changed according to the five patterns shown in Figure 4. After culture in AIM, the shape of the cells was observed using phase-contrast microscopy. Because formed gels inside the wells of a microplate had curved shapes at their centers, hbmMSC adhered only to the central areas. Thus, we counted cells extending pseudopodia inside the central circular area (2 mmØ) of the gel surfaces.

### 4.6. Evaluation of Adipocyte Differentiation

After three days of culture in AIM, the cell culture medium was changed from AIM to Dulbecco’s modified Eagle medium containing 10% fetal bovine serum and 5 μg/mL insulin as a maintenance medium and additionally cultured for four days at 37 °C. Then, to quantitate adipocyte differentiation after four days of maintenance culture, the AdipoRed^TM^ assay was employed after dialyzing the gels through PBS following the manufacturer’s protocol.

## 5. Conclusions

In this study, we designed PNIPAAm gels using the post-crosslinking method to analyze the effect of repeated dynamic property changes in the gels on hbmMSCs. Through careful evaluation, the gelation method was revealed to be cytocompatible and the repeated changes in the swelling ratios of the PNIPAAm gel affected the shape of hbmMSCs. From the viewpoint of both cytocompatibility and reversibility of changes in mechanical properties, the PNIPAAm gel system could potentially have a great impact on mechanobiology research. Through further modification of the network structure, development of PNIPAAm gels with enhanced temperature-dependent changes in mechanical properties, as well as analyses of the various cellular mechanotransduction events involved during growth in the PNIPAAm gel system, will be possible in the future.

## Supplementary Materials

Supplementary materials can be found at http://www.mdpi.com/1422-0067/19/4/1253/s1.

## Abbreviations

MSC	Mesenchymal stem cell
hbmMSC	Human bone marrow-derived mesenchymal stem cell
PNIPAAm	Poly(*N*-isopropylacrylamide)
LCST	Lower critical solution temperature
PEG	Polyethylene glycol
NHS	*N*-Hydroxysuccinimide
PDMAAm	Poly(*N*,*N*-dimethylacrylamide)
NAPMAm	*N*-3-(Aminopropyl) methacrylamide
MSCBM	Mesenchymal stem cell basal medium
AIM	Adipogenic induction medium
