# Supplementary material for: Mesenchylmal Stem Cell Culture on Poly(N-isopropylacrylamide) Hydrogel with Repeated Thermo-Stimulation
Source: Int J Mol Sci. 2018 Apr 21;19(4):1253. doi: 10.3390/ijms19041253 (PMC5979304; doi:10.3390/ijms19041253)
Supplement: Supplementary file 1 [file ijms-19-01253-s001.pdf]

Supplementary Materials: Mesenchymal Stem Cell Culture on Poly(*N*-isopropylacrylamide) Hydrogel with Repeated Thermo-Stimulation

Aya Mizutani Akimoto, Erika Hasuike Niitsu, Kenichi Nagase, Teruo Okano, Hideko Kanazawa, and Ryo Yoshida

LIVE/DEAD Assay

The cytotoxicity of the PNIPAAm gel was evaluated using the LIVE/DEAD Viability/Cytotoxicity Kit (L3224). The PNIPAAm gel was synthesized by mixing poly(NIPAAm-*co*-NAPMAm), pNHS-*octa*-PEG, and RGDS peptides in the presence of hbmMSCs (2.0×10^5 cells/mL) to encapsulate cells. After seven days of culture in a humidified atmosphere with 5.0% CO_2 at 37°C, the encapsulated cells were double-stained with an acetomethoxy derivative of calcein (for live cells) and ethidium homodimer I (for dead cells) according to the general procedure.

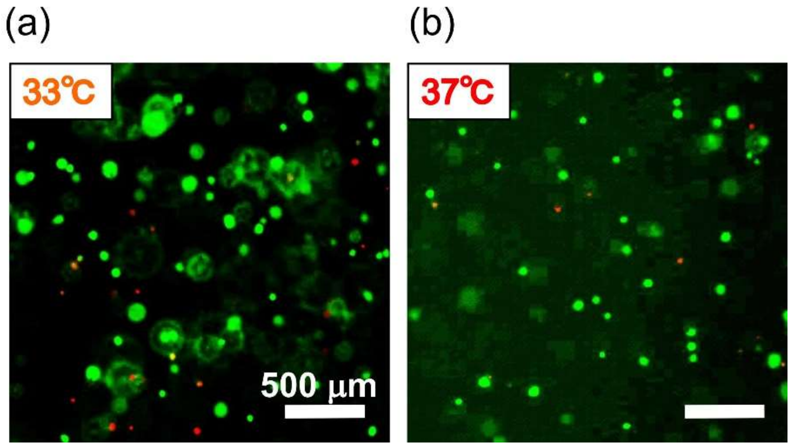

Figure S1. Fluorescence images showing the results of LIVE/DEAD assay for hbmMSCs encapsulated inside the PNIPAAm gel after seven days of culture at (**a**) 33 °C and (**b**) 37 °C.

hbmMSC Culture Using Normal Cell Culture Media

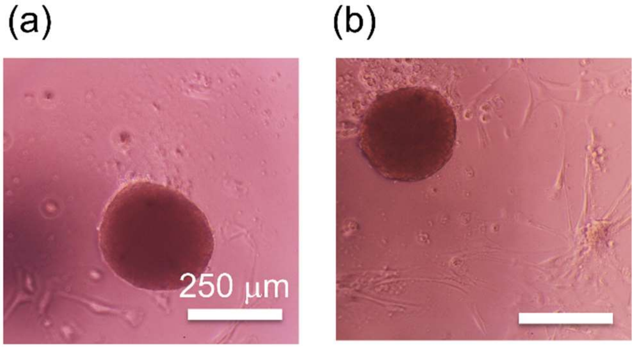

Figure S2. Phase-contrast images of hbmMSCs on the PNIPAAm gel at (a) 33°C and (b) 37°C after four days in culture using MSCBM as cell culture media.

Although the large cell aggregation was observed, all other hbmMSCs showed elongated shapes.
